# Phenotypic Characterization of Creole Cattle in the Andean Highlands Using Bio-Morphometric Measures and Zoometric Indices

**DOI:** 10.3390/ani13111843

**Published:** 2023-06-01

**Authors:** Rolando Rojas-Espinoza, Rassiel Macedo, Alex Suaña, Alfredo Delgado, Yan P. Manrique, Halley Rodríguez, Yesenia M. Quispe, Uri H. Perez-Guerra, Manuel G. Pérez-Durand, Manuel García-Herreros

**Affiliations:** 1 Facultad de Medicina Veterinária y Zootecnia, Universidad Nacional del Altiplano, Puno 21001, Peru; rdrojas@unap.edu.pe (R.R.-E.); yanpierrmvz@gmail.com (Y.P.M.); fhrodriguez@unap.edu.pe (H.R.); mgperez@unap.edu.pe (M.G.P.-D.); 2 Facultad de Ciencias Agrarias, Universidad Nacional San Antonio Abad del Cusco, Cusco 08000, Peru; rassiel.macedo@unsaac.edu.pe; 3 Independent Researcher, Puno 21001, Peru; yulinhor10@gmail.com (A.S.); mayitaz_jessi@hotmail.com (Y.M.Q.); 4 Facultad de Medicina Veterinária, Universidad Nacional Mayor de San Marcos, Lima 15021, Peru; adelgadoc@unmsm.edu.pe; 5 National Institute for Agricultural and Veterinary Research (INIAV), 2005-048 Santarém, Portugal

**Keywords:** bio-morphometric parameters, zoometric indices, Creole cattle, Andean highlands

## Abstract

**Simple Summary:**

The conservation of cattle biotypes as a source of biodiversity that can be used in maintaining their function in different environments is crucial. The present research has been designed to characterize for the first time the bio-morphometric characteristics of the Andean Creole cattle in order to objectively determine their zoometric indices. Based on the obtained results, it was concluded that the Andean Creole cattle biotypes were defined as dual-purpose. Based on this information, it was possible to define the potential productive aptitude of Andean Creole cattle to generate the theoretical basis for future research and genetic characterization.

**Abstract:**

Several Creole cattle biotypes can be found in the Andean highlands, and most of them are considered as being in risk of extinction. The main aim of the present study was to perform a phenotypic characterization of the Creole cattle in the Andean highlands using bio-morphometric measures and zoometric indices. Individuals from three different biotypes (Black ‘Negro’ (n = 57), Colour-Sided ‘Callejón’ (n = 20), and Brindle ‘Atigrado’ (n = 18)) from an experimental research center located in the Peruvian highlands were enrolled in the study. In total, seventeen morphometric parameters were evaluated and ten zoometric indices were calculated in each biotype. To test the relationship between biometric traits, correlation analyses were carried out between morphometric parameters. Differences were observed regarding different morphometric variables such as head length (HL) and rump length (RL) among cattle biotypes (*p* ≤ 0.05). The coefficient of variation (CV; %) regarding different morphometric parameters ranged between 11.32 for neck length (NL) and 3.63 for height at withers (HaW), which indicated low–moderate variability among morphometric variables. Differences were observed in the longitudinal pelvic index (LPI) when different zoometric indices were compared among biotypes (*p* ≤ 0.05). The CV regarding different zoometric indices, which ranged between 10.78 for the cephalic index (CEI) and 5.05 for LPI, indicated low variability among indices. No differences were observed in any other morphometric parameter or zoometric index among cattle biotypes or genders (*p* > 0.05). Finally, multiple correlations were observed between morphometric variables (*p* ≤ 0.05). In conclusion, it was determined that Peruvian Andean Creole cattle can be considered as a dairy-related biotype with a slight tendency for beef production (dual-purpose). The great homogeneity regarding zoometric characteristics among biotypes and genders may indicate that the Andean Creole cattle have been maintained quite isolated, avoiding the genetic influence of other foreign breeds. Finally, the phenotypic characterization including bio-morphometric measurements and zoometric indices obtained from the different Creole bovine biotypes is crucial in order to begin different conservation programs to preserve cattle breeds in the Peruvian Andean highlands.

## 1. Introduction

The first step before the implementation of genotyping tests to better identify different biotypes and breeds in cattle is based on the characterization of phenotypic traits. Two-thirds of the existing cattle in Peru are Creole. Most of the Creole cattle population (around 73.2%) is maintained in the Andean mountains [1]. However, the population of Creole cattle is actually smaller, as mestizo or crossbred cattle are also classified as native Creole cattle. European cattle (*Bos taurus*) were introduced approximately five centuries ago by the Spanish colonization [2]. Thus, up to the present, the Creole cattle have progressively been adapted over multiple generations to extreme environmental conditions and natural pasture feeding [3]. During this period, human–cattle interaction has directly and indirectly influenced the natural selection of Creole cattle, generating new breeds, biotypes, and ecotypes [4].

Creole cattle are essential for human communities living in the Andean region due to their economic and social value, especially in areas of more adverse geography [5]. In this region agricultural activity is crucial. Because of this, Creole cattle have been highly valued for centuries due to their potential as a workforce, based in part on the hardiness that differentiates them from other specialized foreign cattle breeds which have not been able to be adapted to altitude conditions [3]. This hardiness plays a crucial role in several aspects such as resistance to local diseases, high functional longevity, lifespan, and higher fertility rates compared to other more specialized breeds, which makes Creole cattle much more productive in these Andean environments [6,7,8,9].

Nowadays, there is a trend to enhance certain genetic characteristics in livestock marketing. Random crossbreeding has been carried out using more specialized foreign breeds for meat or milk production which resulted in the absorption of the native Creole cattle [3,10,11]. There are regions where Creole cattle have been virtually eliminated due to crossbreeding using other exotic breeds. An example of this is what happened to the Creole cattle of El Salvador which almost completely disappeared [7]. On the other hand, there is little information available related to genetic improvement or management programs applied to Creole cattle [12,13]. This fact together with the lack of information and dissemination of Creole cattle’s productive advantages and economic interest makes the situation more difficult compared to other foreign breeds described in the FAO’s Domestic Animal Diversity Information System (DAD-IS). The lack of attention related to management and selection meant that both the population and the genetic material of Creole cattle have been declining in all Latin American countries [14,15,16].

However, despite the difficulties that Creole cattle are currently facing, they are still considered an important zoo-genetic resource [17,18,19,20]. This is due to the fact that Creole cattle are made up of breeds with differences in productive aptitude, hardiness, and adaptability to climate change [10,21]. Due to the great relevance of Creole cattle as rustic livestock, it is, therefore, necessary to determine the bio-morphometric characteristics and zoometric indices to provide enough technical information in the short term [15]. Zoometry helps to classify an individual based on certain morphological, racial, and productive characteristics in order to establish a specific breed pattern [22]. Such a breed pattern is necessary for species or breed conservation programs which are based on zoometric indices and genomics in order to carry out a specific breed classification [20,23,24,25]. Thus, zoometric studies focus on the morphometric characteristics of the individual according to objective measurements that help to determine the body conformation [26,27]. Therefore, zoometry is necessary to determine the productive aptitude of a given individual from a technical point of view [28]. At present, there is no genetic improvement or well-established preservation programs available for Creole cattle [10]. This could lead to the extinction of this valuable genetic resource [29,30,31].

Due to the above, the main objective of the present study was to characterize for the first time the bio-morphometric characteristics of Creole cattle of the Andean highlands in order to objectively determine their zoometric indices. Based on this information, it would be possible to define the potential productive aptitude of Andean Creole cattle to generate the theoretical basis for future research and genetic characterization.

## 2. Materials and Methods

### 2.1. Ethical Statement

This research was performed in strict accordance with the recommendations in the legal framework (Animal Welfare Law) for all Peruvian Public and Private Laboratories and Higher Education Institutions. The study was conducted according to the guidelines of the Declaration of Helsinki and following the Code of Ethics for animal experiments as reflected in the ARRIVE guidelines available at http://www.nc3rs.org.uk/ARRIVEchecklist (accessed on 1 June 2021). This study was approved by the Bioethics Committee for the use of experimental animals at the Universidad Nacional del Altiplano, Puno, Peru (approval date: 30 June 2020, Code Number: CMTA-019-UNA-CE).

### 2.2. Study Location and Experimental Material

**Figure 1:**
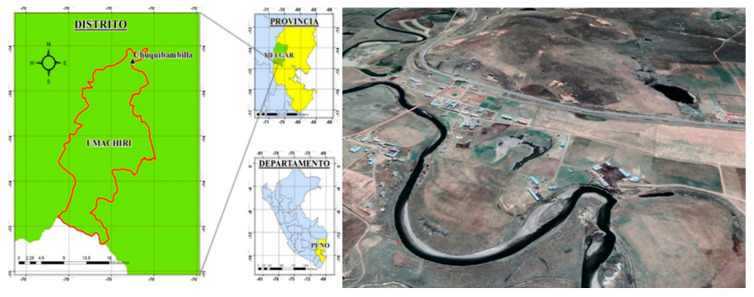
Study area (Andean highlands, Peru): (**Left**): country: Peru; department: Puno (yellow colored region within the blue map); province: Melgar (green colored region within the yellow map); district: Umachiri (green colored region within the green map), area: Chuquibambilla (triangle within the green map). (**Right**): Andean highlands study area in Chuquibambilla Experimental Research Centre within the District of Umachiri (~4000 m.a.s.l.; latitude: 14°47′16.46″ S; longitude: 70°43′42.57″ W).

The study was carried out in the Creole cattle genetic nucleus of the “Chuquibambilla” Research Centre that belongs to the Faculty of Veterinary Medicine and Zootechnics of the National University of the Altiplano of Puno, located in the District of Umachiri, Province of Melgar, Department of Puno, at 3918 m.a.s.l. (latitude: 14°47′16.46″ S; longitude: 70°43′42.57″ W) (Figure 1). The average annual minimum temperature is ~−1 °C, and the maximum is ~28 °C. The average relative humidity is ~79.41% (maximum rainfall during the rainy season: ~85 mm; minimum rainfall during the dry season: ~3 mm) [32].

**Figure 2:**
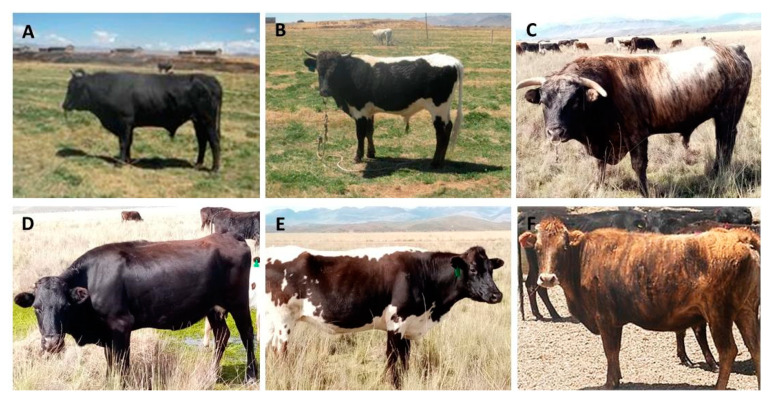
Creole cattle biotypes from the Andean highlands in Peru: (**A**,**D**) Black ‘Negro’ biotype male and female, respectively; (**B**,**E**) Colour-Sided ‘Callejón’ biotype male and female, respectively; and (**C**,**F**) Brindle ‘Atigrado’ biotype male and female, respectively.

The cattle were maintained under an extensive production system using natural pasture feeding. For the study, 12 adult Creole cattle males (four from each biotype) and 83 adult females (53 Black ‘Negro’ biotype, 16 Colour-Sided ‘Callejón’ biotype, and 14 Brindle ‘Atigrado’ biotype) were evaluated (Figure 2). The different biotypes have been bred in the Cattle Experimental Research Centre for more than 30 years. The ancestors from all biotypes were originally sourced from different regions (District of Umachiri, Cupi, Llalli, Macari, and Santa Rosa, among others) in the Province of Melgar. The animals had an average weight of 388.10 kg. The age was determined by dentition observation. Animals with 2 to 8 teeth were included in the study. Adult cows were considered when they had at least one calving. The animals evaluated were free of physical defects to avoid alterations in the bio-morphometric measurements.

### 2.3. Bio-Morphometric Evaluation

The individuals were restrained in a pen for the bio-morphometric evaluation. Different measurements were taken with a tape measure (Anvil, model 96405; Beijing, China) and zoometric sticks. Live weight was calculated with a 1000 kg capacity scale (Mafitel S.A.C., Lima, Peru). The measurements considered were head length (HL), head width (HW), head depth (HD), height at withers (HaW), total length (TL), body length (BL), thoracic perimeter (TP), shank perimeter (SP), abdominal perimeter (AP), thoracic width (TW), thoracic depth (TD), thoracic length (ThL), rump length (RL), rump width (RW), rump height (RH), ischium width (IW), and neck length (NL). All measures were expressed in centimeters [13,14,26,28,33].

### 2.4. Zoometric Indices

Several indices were evaluated to determine the zoometry of the Creole cattle individuals in order to define their ethnological characteristics [14,23,28,33]. The following shows in detail how the different indices were calculated:

- **Cephalic index (CEI)**: (head width/head length) × 100;
- **Thoracic index (TI)**: (thoracic width/thoracic depth) × 100;
- **Body index (BI)**: (body length/thoracic circumference) × 100;
- **Lateral body index (LBI)**: (height at withers/body length) × 100;
- **Anamorphosis index (AI)**: (thoracic perimeter^2^/height at withers) × 100;
- **Pelvic index (PI)**: (rump width/rump length) × 100.

The following indices were used to determine the dairy aptitude:

- **Dactyl-thoracic index (DTI)**: (shank perimeter/thoracic perimeter) × 100;
- **Dactyl-costal index (DCI)**: (shank perimeter/thoracic width) × 100.

The following indices were used to determine the beef aptitude:

- **Transverse pelvic index (TPI)**: (rump width/height at withers) × 100;
- **Longitudinal pelvic index (LPI)**: (rump length/height at withers) × 100.

### 2.5. Statistical Analysis

The database was systematized in a Microsoft Excel sheet (Microsoft Corporation, Redmond, WA, USA). Subsequently, the data were subjected to descriptive statistical analyses to determine the measures of central tendency and dispersion for the respective analysis. The different data from the three biotypes and sex of individuals (male and female) were subjected to assumptions of normality and homoscedasticity and then analyzed by one-way ANOVA in order to identify the potential existence of statistical significance. The ANOVA model included the interaction between biotype and sex (*y =* biotype + sex + biotype*sex *+ e*). Tukey’s test was used to compare means. Finally, Pearson’s correlation was determined to compare pairs of morphometric parameters. All analyses were performed using the R Studio statistical software [34]. The significance level was set at *p* ≤ 0.05).

## 3. Results

### 3.1. Morphometric Assessment of Phenotypic Parameters in Different Creole Cattle Biotypes

Table 1 shows the results obtained from the linear bio-morphometric variables obtained from the different biotypes of Andean Creole cattle. Similar coefficients of variation (CVs) (%) were observed among the different parameters (range: 3.63–11.32). Only the parameters HD, ThL, and NL showed CV > 10%. Significant differences were observed among the different biotypes for HL and RL values (*p* < 0.05). No differences were observed regarding the rest of the bio-morphometric parameters among the different biotypes (*p* > 0.05).

### 3.2. Correlation between Bio-Morphometric Parameters in Creole Cattle

Analysis of all possible correlations found multiple significant positive and negative correlations between morphometric parameters (HL, HW, HD, HaW, TL, BL, TP, SP, AP, TW, TD, TR, RW, RL, RH, IW, and NL) (*p* ≤ 0.001). Strong positive relationships between HL and HaW, AP, and RL were detected (*p* < 0.001), and a strong negative relationship between HD and TD (*p* ≤ 0.01) was observed. No significant correlations were observed between HW and any other morphometric parameter (*p* > 0.05). However, strong positive correlations were detected between HaW and TL, SP, AP, TW, RW, and RL (*p* < 0.001). Moreover, important positive correlations were detected between TL and BL, TP, SP, AP, and RL together with strong positive relationships between BL and TP, AP, and RL (*p* < 0.001). In addition, TP was positively correlated with AP, RL, and RW parameters, and SP was positively correlated with AP and RW (*p* < 0.001). A positive relationship between AP and TW, ThL, and RW was observed, as was a positive relationship between TW and TD, RW, RL, and NL parameters (*p* < 0.001). Strong positive correlations were detected between TD and RW and RL; however, ThL was negatively correlated with IW (*p* < 0.05). Finally, with respect to rump-derived parameters, a positive correlation was observed between RW and RL parameters (*p* < 0.001) (Online Resource Supplementary Table S1).

### 3.3. Zoometric Indices in Different Creole Cattle Biotypes

The zoometric indices obtained from the different biotypes of Andean Creole cattle are shown in Table 2. In general, low CVs were observed (range: 5.05–10.78), with the lowest CV% for LPI and the highest for CEI and DCI (>10%). Significant differences were observed among the different biotypes for LPI index value (*p* < 0.05). No differences were observed in the rest of the zoometric indices among the different biotypes (*p* > 0.05).

### 3.4. Gender-Related Zoometric Indices in Different Creole Cattle Biotypes

The results regarding the zoometric indices related to gender and biotype are shown in Table 3. No significant differences were observed between genders or among biotypes regarding the different zoometric indices (*p* > 0.05). Overall, it was observed that the values of the zoometric indices were greater in males when compared to those in females, with the exception of AI, LPI, TPI, and DCI which were greater in the females belonging to any of the three biotypes although without showing significant differences (*p* > 0.05).

## 4. Discussion

The present study defined for the first time the phenotypic characteristics of three different Creole cattle biotypes in the Andean highlands using bio-morphometric measures and zoometric indices. The statistics of linear variables according to biotype showed similar coefficients of variation (CVs), which demonstrates that morphometric and linear parameters in Creole cattle of the Andean highlands have defined and very homogeneous phenotypic characteristics (only HD, ThL, and NL parameters showed a CV > 10%). This fact may be due to the breeding and selection management scheme that is routinely carried out in the experimental research center where the measure values were taken, as well as to genetic factors inherent to the previous selection of the different biotypes [35]. Thus, it could be determined that Creole cattle maintained in the Andean highlands have not been influenced by other foreign breeds, being considered isolated biotypes [36]. It should be noted that the number of individuals was lower in some experimental groups than in others, and therefore, the results obtained might differ if the number of animals per group was increased. In the present study, the bio-morphometric values obtained were slightly higher than those reported in a previous study under the same conditions by Rojas and Gómez [13]. Among the Black ’Negro’, Colour-Sided ‘Callejón’, and Brindle ‘Atigrado’ biotypes, differences were only observed in the HL and RL values, while no differences were observed in the rest of the parameters.

Despite the adverse climatic conditions in the Andean highlands and the low-quality pastures for feeding the cattle in this region [13], the results obtained for the different bio-morphometric parameters were superior to those reported in Creole cattle from different environments such as Mexico [4], Ecuador [3], Panama [9], Uruguay [37], Argentina [38], and Peru [35]. However, other studies conducted in other regions such as those reported for Argentine Creole cattle showed similar results to those obtained in the present study with slightly superior values of BL, RW, and RL [8,13], as well as for the Limonero from Venezuela [33] and the Creole Blanco Orejinegro from Colombia [36]. Since the anatomical characteristics of these biotypes have medium–high heritability, this could be improved in specific centers using selection programs as tools for genetic improvement in a few generations [39].

In addition, it can be observed that the live weight was higher than that reported in other studies in Creole cattle, as was the height at withers [3,4,9,35,37]. This fact may lead to defining these animals as medium-sized Creole cattle having a good conformation and a tendency for beef production. At the same time, it can be observed that the thoracic perimeter is higher than that reported by the authors mentioned above, as well as by other authors such as Fernández et al. [38], Contreras et al. [33], and Rabasa et al. [8]. These characteristics of Andean Creole cattle could be due to an adaptation to the hypoxia conditions in the Peruvian Andean highlands as mentioned in previous studies in the same environmental conditions [36]. In the present study, a greater live weight was observed in comparison with the Creole cattle from other countries, which could be due to the genetic characteristics of Andean cattle with a relatively high heritability in this aspect (h^2^ = 0.23 to 0.37). In addition, genetic selection may have increased the cattle body size, which could have been achieved in a few generations by means of genetic improvement programs sustained over time [40].

In the present study, the correlation of live weight with respect to linear variables shows that rump length and rump width have an intermediate positive correlation. This result was similar to that reported in Creole cattle in the Manabi region [41] and also in Creole cattle from Puná Island [11], both in Ecuador. Therefore, the rump morphometric measurements could be used for the prediction of live weight [11,42]. Another interesting result is related to the fact that most of the variables have positive correlations between them, which allows deducing that the Creole cattle from the Andean Altiplano had a harmonious body development during the selection process [36]. Finally, positive intermediate correlations were observed between thoracic perimeter and abdominal perimeter as well as between rump width and rump length, which were also similar to the results reported in Manabi Creole cattle [41] and Teruel Creole [43].

With respect to the zoometric indices obtained, the lowest CV was observed in LPI, and the highest CV was observed in CEI. These indices depend on the relationship among different linear morphometric measurements that allow qualifying the breed based on the body proportion (width vs. length dimensions) in each individual [23].

The indices obtained showed that the ethnological characteristics related to CEI define Creole cattle as dolichocephalic since the head length is greater than the head width, giving an aspect of a long and thin face [12]. The results obtained are similar to those reported by Contreras et al. [33] in Limonero Creole cattle from Venezuela, by Aguirre-Riofrio et al. [3] in Lojano Creole cattle from Ecuador, and also by Rojas et al. [36] in Blanco Orejinegro Creole cattle from Colombia. On the other hand, a higher index was reported in Mixteco Creole bulls from Mexico [14]. Finally, Cabezas et al. [12] and Villalobos-Cortes et al. [9] reported lower indices.

The TI observed shows that Creole cattle of the Andean highlands have a thorax with a tendency to ellipticity. Normally, the lower the TI index value, the more elliptical the thorax, which corresponds to a morphometry related to dairy cattle [37]. Thus, it could be inferred that Andean Creole cattle have dairy aptitude with a predominance of the thorax depth in relation to the thorax width [43]. However, López et al. [14] report greater results in Creole cattle, and Rojas et al. [36] report greater results in Colombian Creole cattle. Similarly, Cabezas et al. [12] observed greater sizes in Santa Elena Creole cattle; greater sizes were also observed by Contreras et al. [33] in Limonero Creole cattle and Villalobos-Cortes et al. [9] in Guaymi Creole cattle from Panama, both considered dairy cattle. Similar TIs were reported in Ecuadorian Lojano Creole cattle [3] and in Uruguayan Creole cattle as well [37].

The BI expresses the relationship between longitudinal diameter and thoracic perimeter defining the Andean Creole cattle body as brevilineal [23]. Therefore, there is a predominance of height over length measurements (shortened body), which is typical in dairy cattle. The results obtained in the present study were lower than those of Ancash Creole cattle [35], Argentine Creole cattle [38], Lojano Creole cattle [3], Uruguayan Creole cattle [37], and Blanco Orejinegro Creole cattle [36]. However, the results obtained are greater than those obtained in Mixteco Creole cattle [14], Limonero Creole cattle [33], and Guaymi Creole cattle [9]. Therefore, it could be considered that the Andean Creole cattle have a brevilinear tendency, with a cannon bone diameter that is even greater than those reported by the authors mentioned above. This characteristic is very important because it gives strength to the animal for grazing, which is considered a typical characteristic of beef cattle [3,23,28].

The LBI (also known as proportionality index) showed that Andean Creole cattle are close to a rectangle shape, as the lower the index value, the more compact the biotype [23,37]. Therefore, the animals enrolled in the present study were individuals with a dual-purpose tendency. Some authors observed greater indices, as in the Mixteco cattle [14] and the Limonero cattle [33], considering them to have a tendency for beef production. Other authors reported lower results, as in the case of the Argentinean cattle [38], considering them to have a compact body-type biotype. In contrast, Guaymi Creole cows were considered dairy cattle because of a greater LBI value [9]. On the other hand, there were authors who had similar results in Ancash Creole cattle [35], Colombian Creole cattle [36], and Uruguayan Creole cattle [37].

Regarding AI index value, the Andean Creole cattle tend to have greater dairy aptitude (ranging from 2.5 to 3) compared to Mixteco cattle [14], Lojano cattle [3], and Uruguayan cattle [37]. On the other hand, similar indices were observed compared to Limonero cattle [33] and Argentinean cattle [38]. With regard to PI index value, the Andean Creole cattle could be considered brachypelvic (the pelvis is slightly longer than wide). This is because the longer and wider the rump, the greater the amount of meat will produce. Moreover, the rump width has been an important parameter for determining reproductive characteristics such as calving ease [3,23,36]. Some authors reported lower indices in Mixteco [14], Lojano [3], Guaymi [9], and Blanco Orejinegro Creole cattle [36], while greater indices were reported in Limonero [33], Argentine [38], and Uruguayan Creole cattle [37].

The DTI and DCI indices have been considered for defining dairy aptitude. The first one indicated a thin skeleton [37] due to the fact that the beef aptitude considers values >11. In this case, the DTI index value obtained was <10, and therefore, the Andean Creole cattle could be defined as dairy-aptitude livestock. The observed DCI index value indicated that there was a tendency for milk production [41]. On the contrary, beef cattle show lower DCI index values. Therefore, it can be inferred that Andean Creole cattle have a clear tendency for dairy production, as several authors stated that dairy aptitude was more related to the DTI index value than to the DCI index value [9,33,36,41]. However, other authors reported similar DTI and DCI indices for other different Creole cattle, and therefore, these cattle tended to have a dual-purpose biotype [3,14].

Finally, the TPI and LPI indices have been considered for defining beef aptitude. In the present study, the obtained values for both indices indicated a certain tendency for beef aptitude (values >33) [23]. There are reports of lower values for these indices in Mixteco Creole cows considered as dual-purpose cattle but with a tendency for beef aptitude [14], as well as in Lojano [3] and Guyami Creole cattle [9]. On the contrary, greater TPI and LPI values were observed in Limonero [33] and Blanco Orejinegro Creole cattle [36].

## 5. Conclusions

In conclusion, the zoometric indices obtained in Andean Creole cattle from the Peruvian highlands indicated that TI, BI, AI, DTI, and DCI values have a tendency for milk production, while LBI, PI, TPI, and LPI values have a tendency for meat production. Therefore, it could be determined that Andean Creole cattle mainly have a dairy-aptitude biotype with a slight tendency for meat production. Therefore, it could be concluded that the Andean Creole biotypes can be defined as dual-purpose cattle but with a dairy tendency. The observed thoracic perimeter was greater than those mentioned in other reports, which could be related to an adaptation to the geographical conditions of the Peruvian Andean highlands. Finally, it would be necessary to complete the present phenotypic study in the future by implementing genotyping tests to better identify the different Andean Creole cattle biotypes from the Peruvian highlands.

## Figures and Tables

**Table 1:** Morphometric parameters obtained from different biotypes of Creole cattle from the Peruvian Andean highlands.

Morphometric Parameters	Biotype				
	Black ‘Negro’	Colour-Sided ‘Callejón’	Brindle ‘Atigrado’	Mean ±S.E.M.	CV (%)
Head length (HL)	50.70 ± 0.42 ^a^	51.69 ± 0.66 ^a,b^	53.14 ± 0.84 ^b^	51.30 ± 0.34	6.02
Head width (HW)	23.98 ± 0.37	23.06 ± 0.35	24.00 ± 0.47	23.81 ± 0.26	9.83
Head depth (HD)	29.02 ± 0.44	28.50 ± 0.66	29.86 ± 0.82	29.06 ± 0.34	10.56
Height at withers (HaW)	124.02 ± 0.63	124.19 ± 1.00	124.50 ± 1.37	124.13 ± 0.49	3.63
Total length (TL)	200.15 ± 2.16	208.13 ± 2.16	200.21 ± 4.27	201.70 ± 1.63	7.37
Body length (BL)	145.49 ± 1.29	147.88 ± 1.68	144.43 ± 3.29	145.77 ± 1.04	6.49
Thoracic perimeter (TP)	176.57 ± 0.97	180.81 ± 1.56	177.29 ± 3.23	177.51 ± 0.88	4.53
Shank perimeter (SP)	17.98 ± 0.12	18.56 ± 0.43	18.36 ± 0.61	18.16 ± 0.15	7.52
Abdominal perimeter (AP)	199.96 ± 1.44	204.63 ± 2.56	204.36 ± 4.59	201.60 ± 1.3	5.87
Thoracic width (TW)	39.11 ± 0.44	39.56 ± 1.32	39.00 ± 1.07	39.18 ± 0.41	9.61
Thoracic depth (TD)	67.19 ± 0.43	67.56 ± 1.82	68.79 ± 0.90	67.53 ± 0.47	6.28
Thoracic length (ThL)	52.51 ± 0.76	51.94 ± 1.77	54.79 ± 1.25	52.78 ± 0.63	10.92
Rump width (RW)	45.94 ± 0.43	48.00 ± 0.98	46.07 ± 0.93	46.36 ± 0.37	7.33
Rump length (RL)	48.00 ± 0.39 ^a^	49.88 ± 0.42 ^b^	48.21 ± 0.93 ^a,b^	48.40 ± 0.31	5.91
Rump height (RH)	125.75 ± 1.93	123.44 ± 0.96	124.57 ± 1.38	125.11 ± 1.27	9.23
Ischium width (IW)	14.43 ± 0.16	14.75 ± 0.42	14.43 ± 0.45	14.49 ± 0.15	9.42
Neck length (NL)	33.51 ± 0.47	30.88 ± 0.96	32.14 ± 1.09	32.77 ± 0.41	11.32

Different superscripts (^a–b^) within files show differences among cattle biotypes (*p* < 0.05). S.E.M.: standard error of the mean; CV: coefficient of variation. Morphometric values are shown in cm.

**Table 2:** Zoometric indices obtained from different biotypes of Creole cattle from the Peruvian Andean highlands.

Zoometric Indices	Biotype				
	Black ‘Negro’	Colour-Sided ‘Callejón’	Brindle ‘Atigrado’	Mean ±S.E.M.	CV (%)
Cephalic index (CEI)	47.42 ± 0.76	44.71 ± 0.79	45.27 ± 1.06	46.53 ± 0.55	10.78
Thoracic index (TI)	58.25 ± 0.61	59.06 ± 2.21	56.74 ± 1.52	58.15 ± 0.63	9.80
Body index (BI)	82.47 ± 0.74	81.88 ± 1.21	81.49 ± 1.21	82.19 ± 0.56	6.20
Lateral body index (LBI)	85.58 ± 0.85	84.11 ± 0.96	86.61 ± 1.53	85.47 ± 0.63	6.68
Anamorphosis index (AI)	2.52 ± 0.03	2.65 ± 0.05	2.52 ± 0.08	2.55 ± 0.02	8.89
Pelvic index (PI)	95.76 ± 0.59	96.32 ± 2.07	95.62 ± 1.18	95.85 ± 0.58	5.48
Dactyl-thoracic index (DTI)	10.20 ± 0.09	10.26 ± 0.21	10.36 ± 0.28	10.24 ± 0.08	7.35
Dactyl-costal index (DCI)	46.27 ± 0.60	47.63 ± 1.74	47.25 ± 1.33	46.7 ± 0.55	10.78
Transverse pelvic index (TPI)	37.06 ± 0.31	38.68 ± 0.84	36.99 ± 0.57	37.36 ± 0.28	6.83
Longitudinal pelvic index (LPI)	38.7 ± 0.26 ^a^	40.21 ± 0.48 ^b^	38.71 ± 0.51 ^a^	38.99 ± 0.22	5.05

Different superscripts (^a–b^) within files show differences among cattle biotypes (*p* ≤ 0.05). S.E.M.: standard error of the mean; CV: coefficient of variation.

**Table 3:** Zoometric indices obtained from different genders (female vs. male) within each biotype of Creole cattle from the Peruvian Andean highlands.

Biotype						
Zoometric Indices	Black ‘Negro’		Colour-Sided ‘Callejón’		Brindle ‘Atigrado’	
	Female	Male	Female	Male	Female	Male
Cephalic index (CEI)	44.71 ± 0.73	47.99 ± 1.30	47.14 ± 1.25	51.86 ± 1.27	46.93 ± 1.15	46.84 ± 1.29
Thoracic index (TI)	54.92 ± 1.13	58.99 ± 1.19	56.31 ± 2.50	58.72 ± 2.44	57.81 ± 1.91	61.43 ± 1.65
Body index (BI)	80.81 ± 1.42	87.67 ± 1.76	86.49 ± 2.24	87.39 ± 3.13	81.64 ± 1.71	89.40 ± 3.15
Lateral body index (LBI)	91.00 ± 1.64	93.97 ± 1.51	86.65 ± 1.52	95.06 ± 3.19	90.55 ± 1.74	92.69 ± 4.24
Anamorphosis index (AI)	2.05 ± 0.05	1.45 ± 0.03	1.98 ± 0.09	1.43 ± 0.01	2.01 ± 0.06	1.45 ± 0.08
Pelvic index (PI)	95.51 ± 1.62	92.80 ± 1.94	92.94 ± 1.84	94.44 ± 2.94	90.54 ± 2.20	98.01 ± 4.00
Dactyl-thoracic index (DTI)	10.35 ± 0.12	11.97 ± 0.28	10.58 ± 0.35	11.79 ± 0.28	10.56 ± 0.21	11.98 ± 0.18
Dactyl-costal index (DCI)	51.46 ± 1.21	49.77 ± 1.13	49.00 ± 3.09	47.77 ± 0.58	47.48 ± 1.54	48.21 ± 2.42
Transverse pelvic index (TPI)	35.10 ± 0.44	30.07 ± 0.56	33.91 ± 0.69	30.17 ± 2.27	33.76 ± 0.84	32.34 ± 1.46
Longitudinal pelvic index (LPI)	36.89 ± 0.57	32.61 ± 1.04	36.51 ± 0.59	31.87 ± 1.41	37.43 ± 0.93	33.03 ± 0.91

Values are shown as mean ± S.E.M. (standard error of the mean). No differences were observed among genders or biotypes (*p* > 0.05).

## Data Availability

All data generated or analyzed during this study are included in this article.

## Supplementary Materials

The following supporting information can be downloaded at: https://www.mdpi.com/article/10.3390/ani13111843/s1, Table S1: Pearson’s (r) correlation indices between different biomorphometric parameters in Creole cattle from the Andean highlands.
