# Supplementary material for: Phenotypic Characterization of Creole Cattle in the Andean Highlands Using Bio-Morphometric Measures and Zoometric Indices
Source: Animals (Basel). 2023 Jun 1;13(11):1843. doi: 10.3390/ani13111843 (PMC10252010; doi:10.3390/ani13111843)
Supplement: Supplementary file 1 [file animals-13-01843-s001.zip › animals-2368250-supplementary.pdf]

Table S1. Pearson´s (r) correlation indices between different bio-morphometric parameters in Creole cattle from the Andean highlands.

	**Head length (HL)**	**Head width (HW)**	**Head depth (HD)**	**Height at Withers (HW)**	**Total length (TL)**	**Body length (BL)**	**Thoracic perimeter (TP)**	**Shank perimeter (SP)**	**Abdom. perimeter (AP)**	**Thor. width (TW)**	**Thor. depth (TD)**	**Thor. length (ThL)**	**Rump width (RW)**	**Rump length (RL)**	**Rump heigth (RH)**	**Ischium width (IW)**	**Neck length (NL)**
**Head length (HL)**	—																
**Head width (HW)**	**0.15**	—															
**Head depth (HD)**	**0.13**	**0.03**	—														
**Height at Withers (WH)**	**0.37*** **	**-0.04**	**0.28****	—													
**Total length (TL)**	**0.33****	**0.08**	**-0.05**	**0.39*****	—												
**Body length (BL)**	**0.24***	**0.09**	**-0.18**	**0.25***	**0.50*****	—											
**Thorac. perimeter (TP)**	**0.29****	**-0.16**	**-0.01**	**0.24**	**0.34*****	**0.42*****	—										
**Shank perimeter (SP)**	**0.21**	**0.08**	**0.05**	**0.30*****	**0.34*****	**0.28****	**0.31****	—									
**Abdom. perimeter (AP)**	**0.41*****	**-0.12**	**0.02**	**0.35*****	**0.45*****	**0.40*****	**0.70*****	**0.36*****	—								
**Thoracic width (TW)**	**0.13**	**0.08**	**0.22***	**0.40*****	**0.16**	**0.02**	**0.09**	**0.19**	**0.21**	—							
**Thoracic depth (TD)**	**0.25***	**0.06**	**0.13**	**0.29****	**0.30****	**0.26****	**0.32****	**0.20**	**0.45*****	**0.40*****	—						
**Thoracic length (ThL)**	**0.09**	**0.07**	**-0.32****	**-0.05**	**0.15**	**0.24****	**0.08**	**0.01**	**0.10**	**-0.15**	**0.29****	—					
**Rump width (RW)**	**0.33****	**0.05**	**0.10**	**0.38*****	**0.29****	**0.28****	**0.43*****	**0.34*****	**0.39*****	**0.44*****	**0.43*****	**0.08**	—				
**Rump length (RL)**	**0.42*****	**0.09**	**0.16**	**0.53*****	**0.46*****	**0.40*****	**0.45*****	**0.33****	**0.47*****	**0.46*****	**0.45*****	**0.00**	**0.68*****	—			
**Rump height (RH)**	**-0.01**	**0.04**	**-0.04**	**0.15**	**0.03**	**-0.02**	**0.11**	**0.07**	**0.13**	**0.32****	**0.10**	**0.04**	**0.16**	**0.11**	—		
**Ischium width (IW)**	**0.20**	**-0.02**	**0.33****	**0.24***	**0.03**	**0.01**	**0.19**	**0.25***	**0.16**	**0.22***	**0.20**	**-0.23***	**0.31****	**0.26****	**-0.02**	—	
**Neck lenght (NL)**	**-0.04**	**0.17**	**0.26****	**0.23***	**0.03**	**0.16**	**-0.09**	**0.14**	**0.01**	**0.39*****	**0.12**	**-0.11**	**0.09**	**0.07**	**0.07**	**0.23***	—

Significance between parameters was shown as * (p≤ 0.05), ** (p≤ 0.01), and *** (p≤ 0.001).
